# Effect of Geographical Access to Health Facilities on Child Mortality in Rural Ethiopia: A Community Based Cross Sectional Study

**DOI:** 10.1371/journal.pone.0033564

**Published:** 2012-03-12

**Authors:** Yemisrach B. Okwaraji, Simon Cousens, Yemane Berhane, Kim Mulholland, Karen Edmond

**Affiliations:** 1 Faculty of Epidemiology and Population Health, London School of Hygiene & Tropical Medicine, London, United Kingdom; 2 Addis Continental, Institute of Public Health, Addis Ababa, Ethiopia; Kenya Medical Research Institute - Wellcome Trust Research Programme, Kenya

## Abstract

**Background:**

There have been few studies that have examined associations between access to health care and child health outcomes in remote populations most in need of health services. This study assessed the effect of travel time and distance to health facilities on mortality in children under five years in a remote area of rural north-western Ethiopia.

**Methods and Findings:**

This study involved a randomly selected cross sectional survey of 2,058 households. Data were collected during home visits to all resident women of reproductive age (15–49 years). A geographic information system (GIS) was used to map all households and the only health centre in the district. The analysis was restricted to 2,206 rural children who were under the age of five years during the five years before the survey. Data were analysed using random effects Poisson regression. 90.4% (1,996/2,206) of children lived more than 1.5 hours walk from the health centre. Children who lived ≥1.5 hrs from the health centre had a two to three fold greater risk of death than children who lived <1.5 hours from the health centre (children with travel time 1.5–<2.5 hrs adjusted relative risk [adjRR] 2.3[0.95–5.6], travel time 2.5–<3.5 hrs adjRR 3.1[1.3–7.4] and travel time 3.5–<6.5 hrs adjRR 2.5[1.1–6.2]).

**Conclusion:**

Distance to a health centre had a marked influence on under five mortality in a poor, rural, remote area of Ethiopia. This study provides important information for policy makers on the likely impact of new health centres and their most effective location in remote areas.

## Introduction

Over 50% of deaths in children under five years occur in children who live in sub-Saharan Africa [1]. There have been recent publications describing the epidemiology of child mortality and the effect of access to health services in urban and rural African populations with well functioning research infrastructure [2]–[6]. However, there have been few studies which have examined associations between access to health care and child health outcomes in remote areas of Africa. The only data available are imprecise estimates derived from statistical models [7]–[9] and the impact of health system interventions for children under five years in remote African regions with high mortality remains poorly understood.

Studies of the effect of geographical access on child mortality from more densely populated areas also report conflicting results [2]–[4], [10], [11]. Reasons include lack of consistency in measures of geographical access. Some studies report on travel time but others report distance travelled from a health facility and do not include the effect of topographical barriers such as mountains or rivers. Other studies only use straight line distance between health facilities and homes.

Determinants of household wealth and access to health care are also closely linked but no studies appear to have assessed interactions between household wealth and access to health facilities or adjusted for these effects. In Ethiopia, Dabat district is remote, rural, poor, and only has one functioning health centre. The University of Gondar has developed a demographic surveillance site in Dabat district (Dabat Health and Demographic Surveillance Site, Dabat HDSS) and wished to develop capacity in socioeconomic data collection and Geographical Information System (GIS) mapping. This study was designed to assess the effect of travel time and distance to health facilities on child mortality in this remote area of rural Ethiopia. A secondary objective was to assess associations between household wealth and child mortality in remote areas.

## Methods

### Ethical considerations

This study was approved by the Ethical review committees of the University of Gondar and the London School of Hygiene and Tropical Medicine. Informed written consent was obtained from all participants in the study.

### Setting

**Figure 1:**
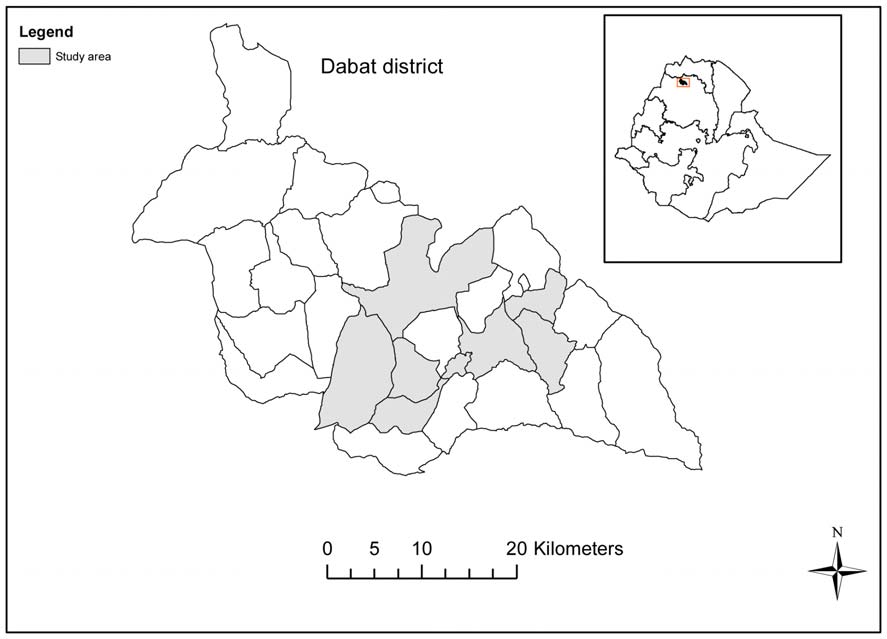
Dabat district and study area.

The study was implemented from January–July 2010 in the Dabat HDSS in Dabat district, north-western Ethiopia (figure 1). The Dabat HDSS consists of three urban and seven rural *kebeles* - the smallest administrative unit in Ethiopia. The population in the Dabat HDSS is currently 46,165 and is dominated by the Amhara ethnic group [12]. A typical house has walls constructed from mud and wood. Livestock are commonly kept in the house and the economy is mainly based on subsistence farming and trading. There are few roads in this part of Ethiopia. Off-road motorised transport is not viable in most areas because of the difficult terrain. The main form of travel is walking and some people live quite close to service centres in terms of distance, but the mountainous region and poor road network means that often the time travelled is high.

**Figure 2:**
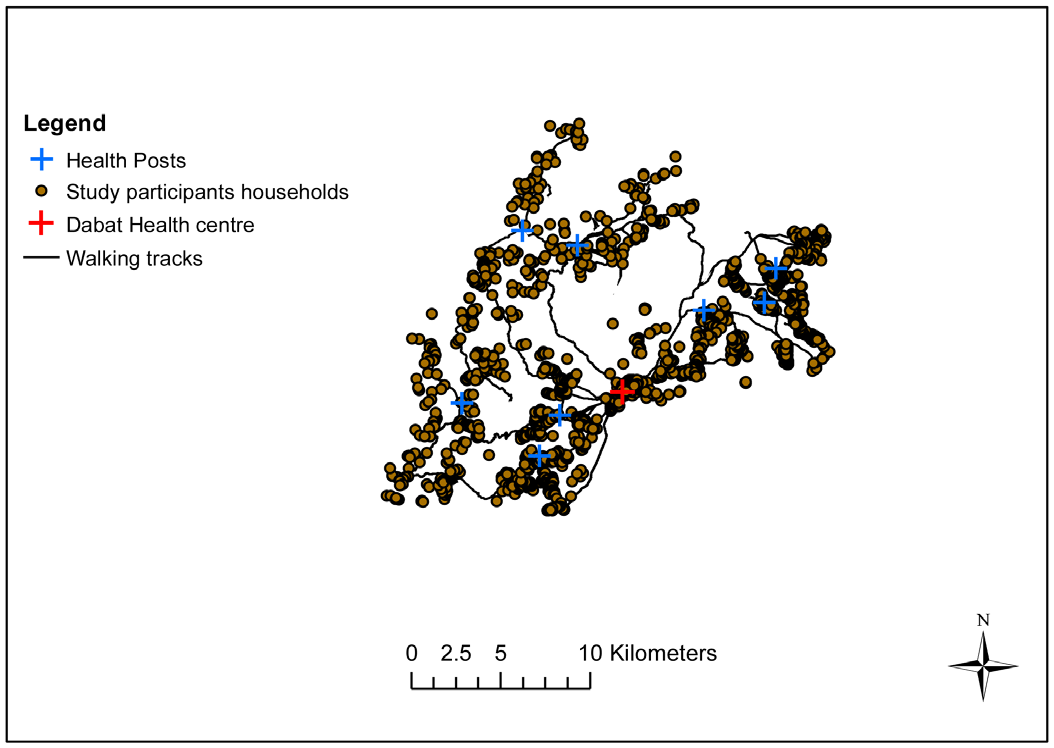
Study households, health posts, health centre and walking tracks in Dabat, rural Ethiopia.

The district health care system comprises Dabat health centre, which is the single functioning health centre. Dabat health centre provides parenteral antibiotics, parenteral oxytocic drugs, parenteral sedatives for eclampsia, manual removal of placenta, manual removal of retained products, anaesthesia and but no caesarean section, blood transfusion, or intensive care facilities such as ventilators or other life support systems. There are few other options for curative care within the district. Eight satellite health posts only provide basic services such as vaccines, oral rehydration treatment and anti-malarial drugs (figure 2).

### Study design

This was a cross-sectional study. Households were selected randomly using a random number generator from the three urban and seven rural *kebeles* in Dabat HDSS. Each household was visited by trained data collectors between January 2010 and July 2010. Only rural *kebeles* were used for the analyses in this study. All women were eligible for inclusion if they were aged between 15 and 49. The analysis was restricted to children who were under five years of age.

### Data collection

Data collectors were recruited from each *kebele* and trained for two weeks. All were high school graduates. Data collectors were trained to visit each household on their list and to continue visiting the household until they had identified a key informant (preferably the head of the household) for each household. Each key informant was asked about the number of women of reproductive age in the household. Each woman of reproductive age was then interviewed separately by the data collector. Each woman was asked about her full birth history - any live births and child deaths she had experienced until the time of survey. Other information recorded included characteristics of mothers and the household asset information needed for the construction of wealth terciles. Data were collected on handheld computers (personal digital assistants, PDAs) using electronic questionnaires with Epihandy software (www.openXdata.org). To validate the accuracy of the data collected, 5% of mothers were re-interviewed by the supervisors.

### GIS data and travel time

The locations (x–y coordinates) of Dabat health centre and each household were first recorded on Geographical Positioning System (GPS) devices by the trained data collectors. The data collectors then followed the same route as study participants and tracked and registered the actual distance travelled (in kilometres) between each village centre and Dabat health centre using the GPS device (figure 2). The straight line distances between the health centre and households were calculated using the “near” function in ArcGIS10 (GIS software package) [13].

Travel time to health centre was calculated using the “Cost analysis” module in the IDRISI Taiga GIS software package [14]. The module requires two input layers of data. The first layer contains the target location (the health centre), and the second layer contains the costs (in terms of travel time) associated with moving through different geographical features in the study area to reach the target feature. Different features (e.g. walking up hills and mountains and traversing through water) are assigned different speeds. The output from the module is an image where each cell (pixel) in the image contains values of travel time required to traverse from that cell to the health centre. In this study, a speed of 5 km/hr was assigned for all walking routes, slopes greater than 30 degrees were assigned a speed of 0.1 km/hr and traversing through water bodies was also assigned a speed of 0.1 km/hr. Travel time for each household was extracted and exported into Stata to merge with the main dataset. We also validated the modelled travel time. We collected actual travel time from 40 village centres in the study area. These data were considered to be the reference values and they were compared with the modelled travel time.

### Statistical methods

All analyses were performed using the survival-time family of commands in Stata SE 11.0 (StataCorp LP, College Station, TX 77845, USA). The primary outcome of interest was under five mortality. We decided a priori that travel time was the most important measure of geographic access because it incorporates the effect of topographical barriers such as mountains and rivers. All analyses were conducted using travel time as the main exposure variable.

The cumulative risk of death at 1 month, 11 months and 59 months was calculated. Under five mortality rates were calculated using deaths as the failure event and person-years of observation as the denominator. Observation time for each child was defined from the time they were born in the five year period before the survey. Observation time ended at the time of death or at the time of interview, whichever occurred first.

A three-level random effects Poisson regression model was initially fitted to account for the intra cluster correlation of many children from the same mother, many mothers from the same households and many households from the same village. However, there was only evidence of within-mother clustering of child deaths (p value<0.0001) and no evidence of clustering at the village (p value = 0.999) or household (p value = 0.145) level. Thus, the final model only adjusted for clustering at the mother level.

We also assessed the effect of important confounding variables (household wealth, mother's education, mother's age, parity, child's sex and distance to the nearest *kebele* health post). We included household wealth and mother's education a priori in the first model (model 1). A second model (model 2) was also developed and included household wealth and mother's education, as well as all confounding factors which had p value of <0.1 for their association with geographic access. To estimate household wealth, we constructed an asset index based on data collected on housing material (walls, floor, windows, and roof) and household assets. The index was constructed using principal component analysis (PCA) in Stata.

## Results

### Study population

2,058 households were randomly selected, 29.4% (605) were urban and 70.6% (1,453) were rural. All households were visited and all women in these households were able to be interviewed. There were 905 women of reproductive age in the rural households and these women reported that that they had 2,206 children younger than five years in the five years before the survey. In the rural households the mean number of children per woman was 4.7 (standard deviation [sd] 2.1) and the mean age of the women was 31 years ([sd] 7.1) (table 1). The women were very homogeneous in religion (99.0% [896/905] Orthodox Christians) and in ethnicity (100% [905/905] Amhara ethnic group). 87.0% (1,925/2,206) of children were born to women with no education. 9.5% (210/2,206) of children lived within one and half hour walking travel time from the health centre and 90.4% (1,996/2,206) of children lived more than one and half hours away (table 1).

**Table 1:** Association between child mortality and explanatory variables in children under age 5 in Dabat, rural Ethiopia.

	Categories	Number of children (%)	Number of deaths	Person-years	Deaths per 1,000 person-years	Crude rate ratio (95% CI)
Child's sex	Boys	1,108(50.2)	79	2604	30	1
	Girls	1.098(49.7)	79	2694	29	1.03(0.75–1.4)
Travel time to the health centre	0–<1.5 hrs	210(9.5)	6	483	12.4	1
	1.5–<2.5 hrs	628(28.4)	42	1515	27.7	2.2(0.93–5.4)
	2.5–<3.5 hrs	624(28.3)	54	1491	36.2	2.9(1.2–6.9)
	3.5–<6.5 hrs	744(33.7)	56	1808	30.9	2.5(1.1–5.9)
	Mean(sd)	2.75 hrs(1.1)				
Distance travelled to the health centre	0–<4 km	178(8.1)	4	416	9.5	1
	4–<8 km	590(26.7)	41	1432	28.6	3.0(1.1–8.5)
	8–<12 km	683(30.9)	54	1620	33.3	3.5(1.3–9.9)
	12–<21 km	755(34.2)	59	1828	32.3	3.4(1.2–9.5)
	Mean(sd)	9.3 km(3.7)				
Straight-line distance to the health centre	0–<3 km	187(8.5)	8	443	18.0	1
	3–<5 km	628(28.4)	45	1506	29.8	1.7(0.77–3.6)
	5–<8 km	642(29.1)	47	1542	30.4	1.7(0.79–3.7)
	8–<15 km	749(33.9)	58	1806	32.1	1.8(0.84–3.9)
	Mean(sd)	6.2 km(2.7)				
Travel time to the health posts	0–<1/2 hr	796(36.1)	59	1937	30.4	1
	1/2–<1 hr	819(37.1)	56	1955	28.6	0.94(0.64–1.4)
	1–<3 hrs	591(26.8)	44	1406	31.2	0.99(0.66–1.5)
Distance travelled to Health posts	0–<2 km	301(13.6)	22	3045	30.4	1
	2–<4 km	966(43.8)	67	2884	28.8	0.95(0.57–1.5)
	4–<12 km	939(42.5)	70	3105	31.0	1.0(0.61–1.6)
Straight-line distance to Health posts	0–<2 km	825(37.4)	56	2751	27.7	1
	2–<4 km	836(37.8)	64	3257	32.5	1.1(0.79–1.7)
	4–<8 km	545(24.6)	39	2962	29.6	1.0(0.69–1.6)
Mother's education	Education	281(12.7)	25	645	38.7	1
	No education	1,925(87.3)	133	4652	28.6	0.71(0.45–1.1)
Mother's age	16–<25 y	619(28.0)	33	1497	22.0	1
	25–<35 y	820(37.1)	70	1965	35.6	1.6(1.1–2.6)
	35–<40 y	394(17.8)	28	945	29.6	1.3(0.79–2.3)
	40–<50 y	373(16.9)	27	889	30.3	1.3(0.80–2.3)
	Mean(sd)	31(7.1)				
Parity	1–3	380(17.2)	23	940	24.4	1.3(0.74–2.2)
	4–5	655(29.6)	64	1534	41.7	2.3(1.4–3.5)
	5–6	711(32.2)	33	1742	18.9	1
	7–13	460(20.8)	38	1081	35.1	1.9(1.1–3.0)
	Mean(sd)	4.7(2.1)				
Household wealth	Least poor	648(29.3)	42	1549	27.1	1
	Middle	758(34.3)	53	1828	28.9	1.1(0.69–1.6)
	Poorest	800(36.2)	63	1921	32.7	1.2(0.81–1.8)

### Geographic access

There was a strong correlation between travel time and straight line distance (correlation coefficient(r) = 0.63, p value< = 0.0001) and distance travelled (r = 0.82, p value< = 0.0001). Table 2 displays the relationship between travel time and important potential explanatory variables. Mothers with no education were more likely to live in a household located greater than 1.5 hours travel time from the health centre compared to mothers with any education (risk ratio [RR] = 1.04, 95% confidence interval [1.01–1.09], p value = 0.044). However, there were no obvious associations between household wealth and geographic access (table 2). Mothers who lived in the poorest households had similar probability of living more than 1.5 hours travel time from the health facility compared to mothers who did not live in the poorest households (RR = 0.99 [0.96–1.02] p value = 0.381).

**Table 2:** Association between travel time and explanatory variables in Dabat, rural Ethiopia.

	Categories	Travel time				P-value
		0–<1.5 hrs	1.5–<2.5 hrs	2.5–<3.5 hrs	3.5–<6.5 hrs	
		Number (%)	Number (%)	Number (%)	Number (%)	
Child's sex	Boys	115(10.4)	310(28.2)	309(28.1)	365(33.2)	0.513
	Girls	95(8.5)	318(28.7)	316(28.5)	378(34.1)	
Mother's education	Education	36(12.8)	96(34.1)	110(39.1)	39(13.8)	<0.0001
	No education	174(9.0)	532(27.6)	514(26.7)	704(36.5)	
Mother's age	16–<25 y	62(10.0)	200(32.3)	172(27.8)	184(29.7)	<0.0001
	25–<35 y	64(7.8)	194(23.6)	230(28.0)	332(40.4)	
	35–<40 y	32(8.1)	125(31.7)	102(25.8)	135(34.2)	
	40–<50 y	52(13.9)	109(29.2)	120(32.1)	92(24.6)	
	Mean(sd)	31(7.1)				
Parity	1–3	38(10.0)	125(32.9)	95(25.0)	122(32.1)	0.003
	4–5	49(7.4)	169(25.8)	179(27.3)	258(39.3)	
	5–6	84(11.8)	201(28.3)	202(28.4)	223(31.4)	
	7–13	39(8.4)	133(28.9)	148(32.1)	140(30.3)	
	Mean(sd)	4.7(2.1)				
Household wealth	Least poor	67(10.3)	154(23.8)	190(29.3)	236(36.4)	0.023
	Middle	61(8.0)	220(29.0)	219(28.8)	258(34.0)	
	Poorest	82(10.2)	254(31.7)	215(26.8)	249(31.1)	

### Child mortality and explanatory variables

**Figure 3:**
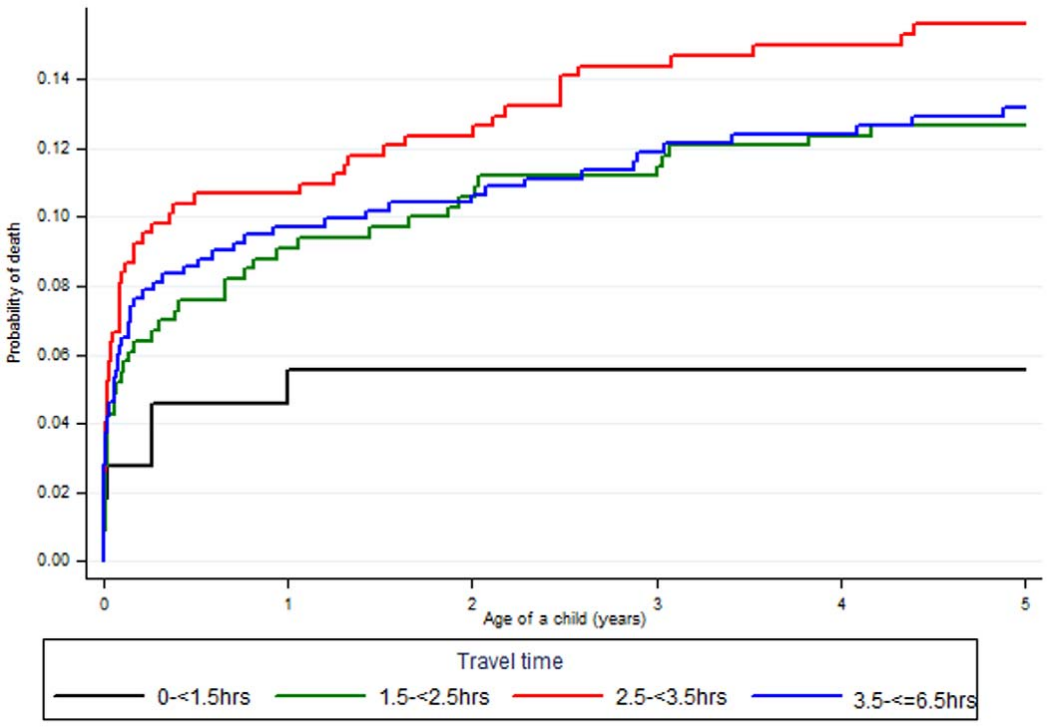
Kaplan Meier cumulative risk of death for children younger than 5 years of age, stratified by travel time in hours to the health centre in Dabat, rural Ethiopia.

There were 158 deaths in the 2,206 children under five years of age included in the study. Of these, 44.0% (69) deaths occurred in the neonatal period (<1 month), 28.0% (45) in the post neonatal period (1–11 months) and 28.0% (44) in children aged 12–59 months. The risk of death for infants aged under one year (1q0) was 93.5 per 1,000 live births (95% CI: 78–111) and for children under five years (5q0) 130 per 1,000 live births (95% CI: 112–150) (figure 3). A total of 5297.5 person-years were observed in the 2,206 children included in the study resulting in a mortality rate of 38 per 1,000 person-years for the five year period prior to the survey.

Children born to educated women had a higher risk of death than children born to women without education, however this result was not statistically significant (RR = 0.71 [0.45–1.1]). Young mothers aged 16–<25 years had a lower risk of child death compared to older mothers. Women with 5–6 children had a lower risk of child death compared to women with fewer children. However, there was no obvious association between household wealth and child mortality. There was also no obvious association between distance to the smaller health facilites (health posts) and child mortality (table 2).

### Child mortality and geographic access

**Figure 4:**
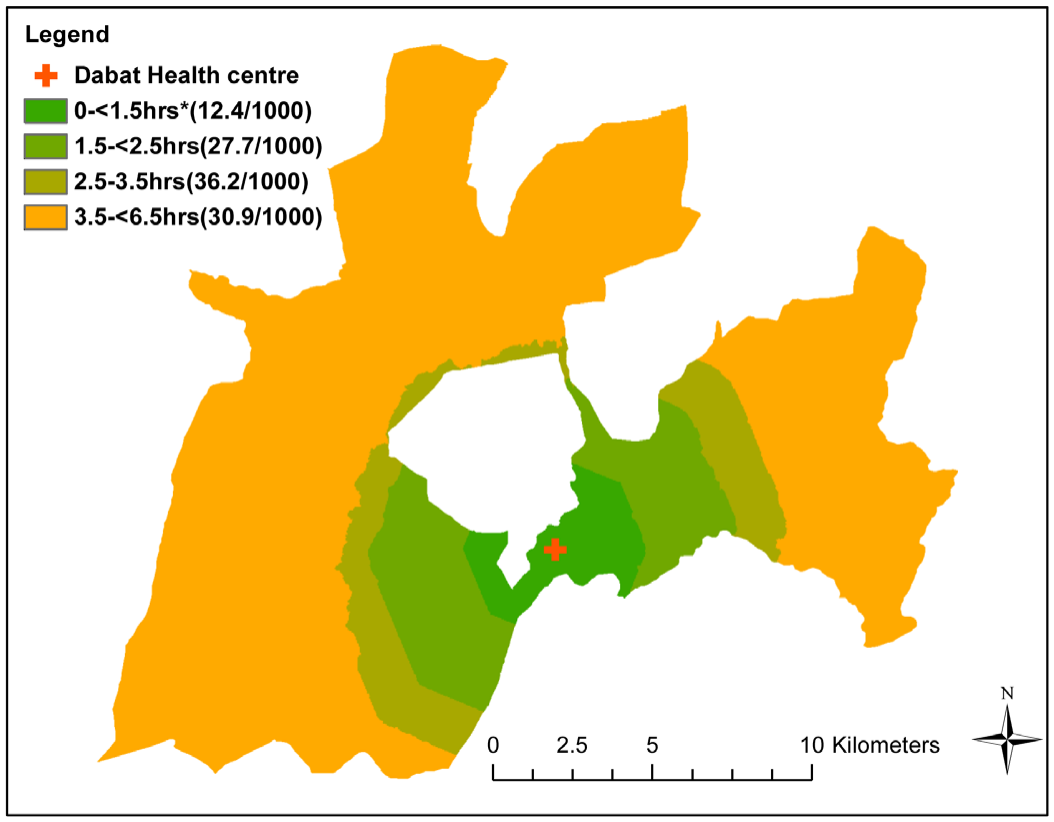
Under 5 mortality per 1,000 person-years by travel time in Dabat, rural Ethiopia.

Table 3 and figure 4 show the relationship between child mortality per 1000 person-years and travel time. Children who lived ≥1.5 hrs from the health centre had a two–three fold greater risk of death than children who lived <1.5 hours from the health centre (children with travel time 1.5–<2.5 hrs adjusted relative risk [adjRR] = 2.3[0.95–5.6], travel time 2.5–<3.5 hrs adjRR = 3.1[1.3–7.4] and travel time 3.5–<6.5 hrs adjRR = 2.5[1.1–6.2]). Our findings were similar when the effect of distance in kilometres to the health centre on child mortality was assessed (table 3). Children living 4–8 km from the health centre (adjRR = 3.6[1.2–10.0]) and children living 8–12 km from the health centre (adjRR = 4.0[1.4–11.4]) had a fourfold greater risk of death compared to children living within four kilometres from the health centre. However, there was no evidence of an association between straight-line distance to the health centre and under five mortality (table 3). Adjusting for mother's education and household wealth (model 1) or maternal age, parity, distance to health posts (model 2) had no substantial effect on the final results. We also found no evidence of an interaction between household wealth and travel time to the health centre (p value = 0.790).

**Table 3:** Association between under 5 mortality and travel time, distance travelled and straight-line distance from the health centre in Dabat, rural Ethiopia.

	Categories	Number of deaths	Person-years	Crude rate ratio (95%CI)	Adjusted rate ratio model 1*	P-value***	Adjusted rate ratio model 2**	P-value***
Travel time (hours)	0–<1.5 hrs	6	483	1	1	0.032	1	0.041
	1.5–<2.5 hrs	42	1515	2.2(0.93–5.3)	2.3(0.9–5.4)		2.3(0.95–5.6)	
	2.5–<3.5 hrs	54	1491	2.9(1.2–6.9)	3.0(1.3–7.1)		3.1(1.3–7.4)	
	3.5–< = 6.5 hrs	56	1808	2.5(1.1–5.9)	2.6(1.1–6.3)		2.5(1.1–6.2)	
Distance travelled (km)	0–<4 km	4	416	1	1	0.023	1	0.016
	4–<8 km	41	1432	3.0(1.1–8.5)	3.1(1.1–8.4)		3.6(1.2–10.0)	
	8–<12 km	54	1620	3.5(1.3–9.9)	3.7(1.3–10.4)		4.0(1.4–11.4)	
	12–< = 21 km	59	1828	3.4(1.2–9.5)	3.7(1.3–10.3)		3.9(1.3–11.0)	
Straight-line distance (km)	0–<3 km	8	443	1	1	0.341	1	0.398
	3–<5 km	45	1506	1.6(0.77–3.6)	1.6(0.77–3.7)		0.9(0.59–1.4)	
	5–<8 km	47	1542	1.7(0.79–3.7)	1.7(0.7–3.7)		1.1(0.69–1.6)	
	8–< = 15 km	58	1806	1.8(0.84–3.9)	1.9(0.8–4.2)		0.9(0.59–1.6)	

* Model 1 adjusted for household wealth and mother's education.

** Model 2 adjusted for all variables in model 1 plus mother's age and parity.

*** P values were calculated using likelihood ratio test.

## Discussion

In a very poor area of remote Ethiopia with a high burden of under-five mortality, we demonstrated that distance from a single functioning health centre was associated with a markedly increased risk of child death. Over 90% of children lived 1.5 or more hours from the health centre. These children had a twofold greater risk of death compared to those who lived within 1.5 hours from the facility.

To our knowledge this is the first study that has examined the relationships between location of health facilities, household wealth and child mortality in such a remote area. Studies of the effect of travel time on child mortality from less remote areas have reported conflicting findings. Some studies indicate that travel time is an important determinant of childhood mortality [2], [15]. However, studies from areas with higher health facility density have demonstrated no evidence of an association [3], [5], [11], [16]–[18]. For example, in Kenya, the lack of association between travel time and child mortality was attributed to high levels of access as most of the households were located within one hour walk from a health facility [19].

In our study, travel time and distance travelled were associated with child mortality but there was no association with straight line distance. Straight line distance simply calculates the shortest distance between two objects (e.g. the household and the health facility). However, people in both urban and rural environments almost never travel straight-line and a number of studies have not demonstrated associations between straight line distance and mortality [11], [16], [20]. Distance travelled is a more useful measure but does not include the effect of natural barriers such as mountains and rivers and the differences in speed of travel through these barriers. ‘Actual’ travel time recorded by trained workers who follow families as they travel to health facilities is the best measure of assess but is costly, time consuming and often not feasible in a resource limited setting. The simpler method of modelled travel time which combines knowledge of road networks, topography, and land cover was used in this study. This method has been validated in a number of studies and has produced reliable and consistent results [19], [21]–[23].

Distance to the smaller health facilities (health posts) was not associated with child survival in our setting. The health workers who staff these posts are trained to provide basic preventative care such as vaccines, oral rehydration solution and anti-malarial drugs. However, they are not trained to provide parenteral antibiotics, anticonvulsants, oxygen or other types of acute care. No other studies appear to have examined the effect of health posts on child under five mortality in sub Saharan Africa and further studies are needed especially from remote areas such as Dabat district.

Interestingly, no studies also appear to have examined interactions between geographic access to health facilities and household wealth. We considered that the effect of distance to health facilities was likely to be more important in poor households who have less funds to pay for transport to facilities than richer families. Thus we carefully assessed the role of household wealth in our study area using a locally created asset index and principal components analysis. We were able to separate our population into household wealth terciles yet we did not find any important differences in child mortality or geographic access between the terciles. Including household wealth and other factors such as maternal education, maternal age, and parity in our multivariable models also had little effect on our estimates of effect size. This is likely to be to due to the marked homogeneity in ethnicity, religion, and household wealth in our study population.

There were limitations to our study. Firstly, due to our small sample size, we were not able to investigate how geographic access to health facilities influenced neonatal, infant, and post infant mortality separately. Other studies have reported greater impact of access to health facilities on neonates and young infants than older children [4], [10], [11]. Our neonatal mortality risks were also similar to other Ethiopian and African data illustrating the importance of providing effective health care for these young infants [24], [25]. Secondly, we may have underestimated under five mortality by not including children whose mother had died. These children have a markedly higher risk of mortality than children whose mother is still alive [26]. However, all women resident in our randomly selected houses were visited and full birth histories were obtained from each woman, thus we were unlikely to have missed other births and deaths in our study sample. Mortality risk in our study population was also consistent with, or higher than, other studies from Ethiopia [20], [27], [28]. Thirdly, in our cross sectional study, distance and travel time were assigned at the point at which the mothers were interviewed and there was no ongoing tracking of migration. Thus there may have been some misclassification of exposure status. Finally, we did not assess effects of health facility access on cause specific mortality. The impact of geographic access is likely to be even more important for severe rapidly progressive conditions such as birth asphyxia, cerebral malaria and septicaemia. However, we did not have the resources to conduct in depth interviews with mothers of children who died in this study (e.g. using verbal autopsy questionnaires) or to ascertain the causes of death of children who died in the health centre.

Our study has important implications for policy and program development. Our study indicates that health centres should be provided within 1.5 hours travel time from villages in remote areas in rural Africa. Following a health facility rehabilitation and expansion program, Ethiopia is now increasing the number of health centres in rural and urban areas [29]. Our findings will assist health policy makers in Ethiopia and other countries in sub Saharan Africa to understand the likely impact of health infrastructure schemes and the most effective location of health facilities. Our study also provides important epidemiological information about children living in remote areas. We have reported high mortality risks in a population that has very poor access to health care. There has been only one functioning health centre in the study area for the last 20 years. This is yet another example of the inverse care law, i.e. that the availability of good medical care tends to vary inversely with the need for it in the population served [30]. There has also been no investment in research infrastructure in this remote area until the present time and these resources remain markedly limited. There has also been little funding for research networks and demographic surveillance systems in remote areas in low-income countries. Greater resources, funding, and health research infrastructure must be provided. This is required to develop and evaluate health systems and programs that will reach the children who are most in need.

## References

[1] Black RE, Morris SS, Bryce J (2003). Where and why are 10 million children dying every year?. Lancet.

[2] Schoeps A, Gabrysch S, Niamba L, Sie A, Becher H (2011). The effect of distance to health-care facilities on childhood mortality in rural Burkina Faso.. Am J Epidemiology.

[3] Moisi JC, Gatakaa H, Noor AM, Williams TN, Bauni E (2010). Geographic access to care is not a determinant of child mortality in a rural Kenyan setting with high health facility density.. BMC Public Health.

[4] Målqvist M, Sohel N, Do TT, Eriksson L, Persson LA (2010). Distance decay in delivery care utilisation associated with neonatal mortality. A case referent study in northern Vietnam.. BMC Public Health.

[5] Rutherford ME, Dockerty JD, Jasseh M, Howie SRC, Herbison P (2009). Access to health care and mortality of children under 5 years of age in the Gambia: a case-control study.. Bulletin of the World Health Organization.

[6] Rutherford ME, Mulholland K, Hill PC (2010). How access to health care relates to under-five mortality in sub-Saharan Africa: systematic review.. Trop Med and Int Health.

[7] Bryce J, El Arifeen S, Pariyo G, Lanata C, Gwatkin D (2003). Reducing child mortality: can public health deliver?. Lancet.

[8] Darmstadt GL, Bhutta ZA, Cousens S, Adam T, Walker N (2005). Evidence-based, cost-effective interventions: how many newborn babies can we save?. Lancet.

[9] Claeson M, Gillespie D, Mshinda H, Troedsson H, Victora CG (2003). Knowledge into action for child survival.. Lancet.

[10] Armstrong Schellenberg JRM, Mrisho M, Manzi F, Shirima K, Mbuya C (2008). Health and survival of young children in southern Tanzania.. BMC Public Health.

[11] Becher H, Muller O, Jahn A, Gbangou A, Kynast-Wolf G (2004). Risk factors of infant and child mortality in rural Burkina Faso.. Bulletin of the World Health Organization.

[12] Tadesse T, Demissie M, Berhane Y, Kebede Y, Abebe M (2011). Two-thirds of Smear-Positive Tuberculosis Cases in the Community Were Undiagnosed in Northwest Ethiopia: Population Based Cross-Sectional Study.. PloS One.

[13] ESRI (2011). ArcGIS Desktop: Release 10.

[14] Eastman JR (2009).

[15] Stekelenburg J, Kashumba E, Wolffers I (2002). Factors contributing to high mortality due to pneumonia among under-fives in Kalabo District, Zambia.. Trop Med and Int Health.

[16] Diallo a H, Meda N, Ouédraogo WT, Cousens S, Tylleskar T (2011). A prospective study on neonatal mortality and its predictors in a rural area in Burkina Faso: Can MDG-4 be met by 2015?. Journal of Perinatology.

[17] Dummer TJB, Parker L (2004). Hospital accessibility and infant death risk.. Archives of Disease in Childhood.

[18] Armstrong Schellenberg JR, Nathan R, Abdulla S, Mukasa O, Marchant TJ (2002). Risk factors for child mortality in rural Tanzania.. Trop Med and Int Health.

[19] Noor AM, Amin AA, Gething PW, Atkinson PM, Hay SI (2006). Modelling distances travelled to government health services in Kenya.. Trop Med and Int Health.

[20] Byass P, Fantahun M, Mekonnen W, Emmelin A, Berhane Y (2008). From birth to adulthood in rural Ethiopia: the Butajira Birth Cohort of 1987.. Paediatric and Perinatal Epidemiology.

[21] Brabyn L, Skelly C (2002). Modeling population access to New Zealand public hospitals.. Int J Health Geographics.

[22] Tanser F, Gijsbertsen B, Herbst K (2006). Modelling and understanding primary health care accessibility and utilization in rural South Africa: an exploration using a geographical information system.. Social Science & Medicine.

[23] Tanser F (2006). Methodology for optimising location of new primary health care facilities in rural communities: a case study in KwaZulu-Natal, South Africa.. Journal of Epidemiology and Community health.

[24] Lawn JE, Lee ACC, Kinney M, Sibley L, Carlo W a (2009). Two million intrapartum-related stillbirths and neonatal deaths: where, why, and what can be done?. International Journal of Gynaecol and Obstet.

[25] Lawn JE, Cousens S, Zupan J (2005). 4 million neonatal deaths: when? Where? Why?. Lancet.

[26] Ronsmans C, Chowdhury ME, Dasgupta SK, Ahmed A, Koblinsky M (2010). Effect of parent's death on child survival in rural Bangladesh: a cohort study.. Lancet.

[27] Central Statistical Authority (2006). Ethiopia Demographic and Health Survey 2005.

[28] Central Statistical Authority (2011). Ethiopia Demographic and Health Survey 2011 Preliminary Report.

[29] Federal Ministry of Health (2005). Ethiopia Health Sector Strategic Plan (HSDP- III).

[30] Hart JT (1971). The inverse care law.. Lancet.

